# Genetic evaluation of sudden unexpected death in infants and children

**DOI:** 10.1007/s00431-025-06490-1

**Published:** 2025-10-09

**Authors:** A. M. Pries, S. N. van der Crabben, H. A. Moll, W. P. te Rijdt, K. T. Verbruggen, P. J. Puiman, PESUDIC collaborative, A. Custers, E. Edelenbos, J. Fuijkschot, B. Levelink, P. J. Puiman, B. A. Semmekrot, K. T. Verbruggen, H. Vlaardingerbroek

**Affiliations:** 1 Department of General Paediatrics, Erasmus University Medical Center Sophia Children’s Hospital, Dr. Molenwaterplein, 3015 GD Rotterdam, the Netherlands; 2 Department of Human Genetics, Amsterdam University Medical Center, Amsterdam, the Netherlands; 3 Department of Clinical Genetics, Erasmus University Medical Center, Rotterdam, the Netherlands; 4 Department of General Paediatrics, University Medical Center Groningen Beatrix Children’s Hospital, Groningen, the Netherlands

**Keywords:** SIDS, SUDC, SUDI, SUDY, Postmortem, Child death review, Molecular autopsy

## Abstract

The Dutch Postmortem Evaluation of Sudden Unexpected Death in Infants and Children (PESUDIC) procedure investigates cases of sudden unexpected death in infants and children (SUDI and SUDC). These deaths may have a genetic basis. This national retrospective study examined the yield of genetic testing in PESUDIC cases to provide recommendations for genetic evaluation of SUDI and SUDC. Children who underwent the PESUDIC procedure from 2016 to 2022 and had genetic testing performed were included. They were categorized into three groups: (A) cases where standard PESUDIC investigations yielded contributive findings; (B) cases with no contributive findings from standard investigations, but with comorbidities present; (C) cases without contributive findings from standard investigations or comorbidities. The indication and type of genetic testing were determined based on differential diagnostic considerations. Standardized data on medical history, postmortem diagnostic outcomes, and genetic testing outcomes were collected. Genetic testing was performed in 102 cases (median age 1.1 years, 52% male). Prevalence of (likely) pathogenic (LP/P) variants was 20% in group A (*n* = 66), 27% in group B (*n* = 11), and 28% in group C (*n* = 25). In 13 out of these 20 cases, these variants were considered contributive to the cause of death, involving both cardiac (8%) and non-cardiac pathways (5%).

*Conclusion*: We found a high diagnostic yield from genetic testing in cases of SUDI and SUDC, particularly when routine postmortem examination failed to identify a cause of death. These findings support the usefulness of comprehensive genetic evaluation in cases of unexpected paediatric death.

**Table:** 

**What is Known:** • *Previous studies have identified both cardiac and non-cardiac genetic causes of death for cases with pediatric sudden death.* • *Identifying a genetic cause can have profound implications for family members, such as enabling targeted surveillance and preventive interventions for at-risk family members and informing reproductive decision-making.*	
**What is New:** • *Genetic evaluation of infants and children with sudden unexpected death has a high diagnostic yield, especially when routine postmortem examinations did not identify a cause of death.* • *In a comprehensive postmortem investigation of sudden pediatric death broad genetic testing involving both cardiac and noncardiac pathways should be considered.*

**Supplementary Information:**

The online version contains supplementary material available at 10.1007/s00431-025-06490-1.

## Introduction

In 2016, the Netherlands introduced the Postmortem Evaluation of Sudden Unexpected Death in Infants and Children (PESUDIC) procedure to investigate sudden unexpected deaths in individuals aged 0–18 years [1]. The primary goals of this procedure are to improve understanding of underlying causes of death and to help prevent future child deaths. Previous studies from the PESUDIC cohort have shown that a cause of death can be determined in over 70% of cases, most often through autopsy, followed by microbiological testing [1, 2].

Determining the cause of death in these cases has profound implications, particularly for surviving family members [3]. Some causes of sudden death may have a genetic origin, indicating potentially increased risk of similar fatal events in siblings, parents, and other relatives [4–7]. Identifying a genetic cause can enable targeted surveillance and preventive interventions for at-risk family members, thereby reducing morbidity and mortality. It also opens up options for reproductive decision-making, including options for prenatal genetic testing.

However, genetic testing presents challenges. It is expensive, and results may include variants of unknown significance (VUS), complicating interpretation and potentially leading to misinformed conclusions that may burden bereaved families [8]. Moreover, broad genetic testing can uncover incidental findings that negatively impact family members with unforeseen psychosocial impacts. These complexities warrant a careful, multidisciplinary approach to genetic evaluation in the context of sudden unexpected paediatric death.

Previous studies on genetic testing in paediatric sudden death have mainly focused on identifying genes associated with inherited cardiac conditions such as cardiomyopathies and arrhythmia syndromes [4, 5]. Current clinical guidelines recommend genetic testing for these cardiac conditions in cases where no cause of death is found after autopsy [9–11]. It is estimated that 4–20% of sudden infant death syndrome (SIDS) cases are attributable to inherited cardiac diseases [5, 12]. In cases of sudden unexpected death among children and young adults, the reported frequency of cardiac genetic causes is slightly higher, ranging from 13 to 29% [5, 13, 14]. 

However, the etiology of paediatric sudden death is not limited to the cardiovascular system. Other possible genetic contributors include epilepsy, neurodevelopmental disorders, neuromuscular disorders, inborn errors of metabolism, and multisystem syndromes. Research by Pryce et al. suggested that undetected metabolic disease accounted for approximately 2% of sudden unexpected deaths in infants (SUDI) based on acylcarnitine profiles obtained at autopsy [7]. Similarly, a study by Koh et al. applied a gene panel targeting cardiac, neurological, and systemic/syndromic disease to cases of SIDS and sudden unexpected death in children (SUDC) under 3 years of age. Pathogenic (P), likely pathogenic (LP), or VUS variants with possible pathogenic relevance were identified in 11% of cases, nearly half of which were related to non-cardiac causes [6]. These findings suggest that limiting genetic testing to cardiac-related genes may lead to missed diagnoses. Nonetheless, broad genetic testing also raises ethical and logistical concerns, particularly regarding interpretation, psychological impact, and implications for families and the healthcare system.

The PESUDIC procedure has established a cohort of pediatric sudden death cases that underwent standardized postmortem investigation, including detailed medical history, biochemical and microbiological testing, whole-body imaging, and autopsy [1]. Genetic testing was performed selectively, based on clinical indication derived from these investigations and with parental consent. This study aims to assess the genetic yield of (likely) pathogenic variants contributing to the cause of death within the PESUDIC cohort and offers recommendations for integrating genetic testing into the evaluation of SUDI and SUDC cases.

## Methods

This retrospective observational study analysed data from children who underwent the Dutch PESUDIC procedure. The study was exempt from full review by the Medical Research Ethics Committee of the Erasmus University Medical Centre Rotterdam (reference MEC-2021-0421), and the requirement for additional informed consent was waived. However, parental consent is a condition for participation in the PESUDIC procedure as a whole, as well as for the respective elements, and the use of resulting data for research purposes. As such, not all children underwent the full range of postmortem investigations.

### Study population

The PESUDIC procedure, previously described in detail, is applied to cases involving children aged 0–18 years whose death is classified by a forensic medical examiner as natural and unexpected, yet without a clear cause [1]. Death is considered unexpected if the child was previously healthy, had a stable chronic condition, or suffered from an acute illness not anticipated to result in death. The timing of symptom onset is not a limiting factor for inclusion. Stillbirths are excluded from participation [1]. Comorbidity was defined based on medical history using the Pediatric Medical Complexity Algorithm and included prematurity and/or birthweight below the 10th percentile for gestational age [16]. For this study, all PESUDIC cases from 2016 through 2023 were included if parental consent for research use was obtained and genetic testing had been performed.

### Data collection

The PESUDIC protocol includes a standardized sequence of postmortem investigations: medical history review, physical examination, biochemical and microbiological testing, whole-body imaging, and autopsy. Genetic testing is not part of the routine PESUDIC protocol but was conducted selectively in cases where the history or postmortem findings suggested a potential underlying genetic disorder. The indication and type of genetic testing were based on differential diagnostic considerations by the clinical geneticist and the multidisciplinary audit, and therefore varied between cases.

Data on genetic testing and outcomes were obtained from the national PESUDIC database and supplemented by genetic reports in patient records. The PESUDIC database contains standardized information on clinical history, circumstances of death, diagnostic investigations, and outcomes.

Deoxyribonucleic acid (DNA) was extracted from postmortem samples, including whole blood, fibroblasts, or other tissues obtained during autopsy. Genetic analyses were performed in accredited diagnostic laboratories using established protocols. Depending on the suspected pathology, testing included targeted gene analysis, whole exome sequencing (WES), mitochondrial DNA sequencing, or gene panels based on either next-generation sequencing (NGS) or WES platforms.

### Outcome measures

Cause of death was determined during a multidisciplinary audit involving experts in general paediatrics, forensic medicine, radiology, pathology, clinical genetics, paediatric cardiology, infectious diseases, metabolic disorders, and SIDS. Each case was categorized as having an explained, plausible, or unexplained cause of death, according to previously established criteria [1].

- Explained: A death was classified as explained when a diagnostic finding was identified that, by itself, was sufficient to account for the death, and/or was consistent with the clinical history.
- Plausible: A death was classified as plausible when there was a diagnostic finding, but it did not fully align with the clinical history or observed symptoms. Alternatively, a death was also considered plausible when the clinical history was strongly suggestive of a specific cause—such as a witnessed cardiac arrest with features indicative of an arrhythmic event—but no definitive evidence was found during postmortem investigations to confirm the suspected diagnosis.
- Unexplained: A death remained unexplained when neither the clinical history nor postmortem investigations revealed findings that could plausibly account for death.

For this study, explained and plausible cases were grouped as having a determined cause of death. Diagnostic tests, including genetic testing, were considered contributory when their results supported the final determination of the cause of death. Multiple diagnostic modalities could be contributory in a single case.

Genetic variants were classified as pathogenic (P), likely pathogenic (LP), variants of uncertain significance (VUS), or benign, in accordance with the guidelines of the American College of Medical Genetics and Genomics and the Association for Molecular Pathology (ACMG/AMP) [15].

To address the heterogeneity in indications for postmortem genetic testing and allow for a structured comparison, the included cases were categorized into three distinct groups:

- Group A: cases in which standard PESUDIC investigations yielded findings that contributed to determining a cause of death.
- Group B: cases without contributive findings from standard PESUDIC examinations, but with a documented comorbidity based on medical history.
- Group C: cases lacking both contributive findings from PESUDIC investigations and any known comorbidity.

### Statistical analysis

Statistical analyses were performed using SPSS 28 for Windows (IBM Corp. Released 2021. IBM SPSS Statistics for Windows, Version 28.0. Armonk, NY: IBM Corp). The data were summarized with descriptive analyses, and differences between population and age groups were analysed using chi-square tests and an alpha level of 0.05.

## Results

Data were available for 275 PESUDIC cases investigated between 2016 and 2023. The median age of the cases was 1.1 years (interquartile range: 0.3–6.1 years), and 101 cases (59%) were boys. Of these, 102 cases underwent postmortem genetic testing. These cases were stratified into the three groups (Table 1).

**Table 1:** Characteristics of the different population groups

	Group A: Contributive findings, ***n*** = 66 (%)	Group B: No findings, comorbidity present, ***n*** = 11 (%)	Group C: No findings, no comorbidity, ***n*** = 25 (%)
Age — years	1.5 (IQR 0.4–11.9)	1.8 (IQR 0.3–5.1)	0.3 (IQR 0.1–0.5)
Sex — male	35 (53)	7 (64)	11 (44)
Comorbidity*	27 (41)	11 (100)	n.a
Pre/dysmaturity	12 (18)	5 (45)	n.a
Diagnosed genetic disorder	2 (3)	2 (20)	n.a
Unspecified developmental disorder	3 (5)	1 (10)	n.a
Symptoms in the day/week prior to death	58 (88)	7 (64)	17 (68)
Fever	27 (41)	3 (28)	4 (16)
Dyspnea or coughing	19 (29)	2 (18)	3 (12)
Vomiting or diarrhea	25 (38)	1 (9)	5 (20)
Convulsion	3 (5)	0 (0)	1 (4)
Other	54 (82)	6 (54)	12 (48)
Medical help-seeking for symptoms	32 (49)	1 (9)	3 (12)
Death during unsupervised/sleep period	32 (49)	9 (82)	22 (88)
Family history of sudden death	9 (14)	2 (18)	7 (28)
PESUDIC examinations performed			
Physical examination	60 (91)	10 (91)	24 (96)
Biochemical analysis	59 (89)	11 (100)	24 (96)
Microbiological analysis	63 (96)	11 (100)	25 (100)
Imaging	62 (94)	10 (91)	22 (88)
Autopsy	49 (74)	5 (46)	17 (68)

*Multiple comorbidities may be present in one case

Contributive findings are findings that support the final determination of cause of death

In the supplementary information online additional details can be found on the specific comorbidities, PESUDIC findings and genetic variants identified in cases of group A and B.

### Patient characteristics

Table 1 summarizes the characteristics of the three groups. The most frequently observed comorbidity in Groups A and B was pre- and/or dysmaturity. Group C consisted of significantly younger cases. Across all groups, most cases exhibited symptoms such as fever or agitation in the day or week preceding death. A family history of sudden death was most frequently reported in Group C (28%), compared to 14% in Group A and 18% in Group B. Nearly all cases underwent the majority of standard PESUDIC examinations, though autopsy rates were lower overall. Particularly in Group B, only 5 cases (46%) underwent autopsy. By definition, all cases in Group A had contributory findings, based on the following diagnostics: history in 48 cases (73%), physical examination in 7 cases (11%), biochemical analysis in 12 cases (18%), microbiological analysis in 23 cases (35%), imaging in 33 cases (50%), and autopsy in 47 cases (71%).

### Genetic testing and outcomes

Table 2 provides details on the genetic tests performed and their outcomes per group. In Group A, gene panels were the most frequently utilized method (65%), with a total of 12 different panels selected; in addition to those for arrhythmias and cardiomyopathies, panels targeting immunodeficiencies were common. WES was performed in 19 cases (29%). A trio/duo analysis was included in 22 cases (33%). In this group, one or multiple genetic variants were identified in 36% of cases. An LP/P variant was found in 14 cases (21%). For 11 cases (17%), the genetic test results were considered contributory to determining the cause of death, 6 of which (55%) were related to cardiac causes (Table 3).

**Table 2:** Genetic testing performed and outcomes per population group

Genetic testing performed*	Group A: Contributive findings, *n* = 66 (%)	Group B: No findings, comorbidity present, *n* = 11 (%)	Group C: No findings, no comorbidity, *n* = 25 (%)
SNP array	17 (26)	5 (46)	8 (32)
Sequencing specific genes	20 (30)	3 (27)	7 (28)
Gene panel	43 (65)	8 (73)	18 (72)
Arrhythmia	15 (23)	4 (36)	13 (52)
Cardiomyopathy	17 (26)	3 (27)	5 (20)
Sudden cardiac death	4 (6)	0 (0)	1 (4)
Multiple congenital anomalies	7 (11)	4 (36)	3 (12)
Immunodeficiency	9 (14)	0 (0)	0 (0)
Metabolic disorders	4 (6)	1 (9)	2 (8)
Mitochondrial disorders	2 (3)	1 (9)	2 (8)
Glycogen storage disease	0 (0)	0 (0)	1 (4)
Epilepsy	1 (2)	0 (0)	1 (4)
Neuronal migration disorders	0 (0)	0 (0)	1 (4)
Coagulation disorders	3 (5)	0 (0)	0 (0)
Noonan/RASopathy	1 (2)	0 (0)	1 (4)
Thoracic aortic aneurysm and dissection	0 (0)	1 (9)	0 (0)
Nephrotic syndrome	1 (2)	0 (0)	0 (0)
Abnormality of the genitourinary system	1 (2)	0 (0)	0 (0)
Macrocephaly/large length	0 (0)	0 (0)	1 (4)
Whole Exome Sequencing	19 (29)	4 (36)	6 (24)
Mitochondrial DNA analysis	4 (6)	0 (0)	2 (8)
Trio/duo analysis	22 (33)	6 (55)	11 (44)
Genetic analysis outcomes			
Likely pathogenic variant**	3 (5)	3 (27)	2 (8)
Pathogenic variant**	11 (17)	0 (0)	5 (20)
Variant of unknown significance**	10 (15)	4 (36)	7 (28)
Cause of death identified	66 (100)	0 (0)	2 (8)
Genetic analysis contributive	11 (17)	0 (0)	2 (8)
Cardiological or genetic screening of family members recommended	33 (50)	7 (64)	14 (54)

*Multiple types of genetic testing and panels could be combined in one case

**Multiple variants could be identified in one case. The number depicted represents the number of cases in which this level of variant was the highest identified for that case

**Table 3:** Genes with variants that were contributive for determining the cause of the sudden death according to testing method

Group	Testing method^a^		Gene	Variant class^c^	ACMG criteria	Age category	Comorbidity	Autopsy	Cause of death
A	Sequencing specific genes		*SLC25A20*	LP	PM1, PM2, PM3, PP3, PP4	<1 year	None	Not performed	Hypoglycemia from CACT deficiency
	Panel	Arrhythmias	*SCN5A*	P	PS2, PS3, PS4, PM1, PM2, PP2, PP3	≥12 years	None	Signs of lymphocytic myocarditis	Arrhythmia following viral myocarditis
		Cardiomyopathy	*DMD*	P	PVS1, PS4, PM2, PM6	<1 year	None	Slightly dilated right ventricular wall and very low proportion of dystrophin in heart tissue	Cardiomyopathy
			*PRKAG2*	LP	PS4, PP1, PM2, PP3	≥12 years	None	Enlarged heart with fibrosis and a prominent right ventricular wall	Arrhythmia
		Multiple congenital anomalies	*HMGCL*	P^b^	PM1, PM2, PP2, PP3, PP5	<1 year	None	Not performed	Hypoglycemia from HMG-CoA lyase deficiency
			*MRPL44*	P^b^	PS3, PM2, PM3, PM5	1–6 years	None	Heavy heart with hypertrophy and degenerative changes	Hypertrophic cardiomyopathy
		Immunodeficiency	*ADA*	P^b^	PVS1, PM2, PM3 and PS3, PM2, PM3, PP4	<1 year	Prematurity	Necrotizing enterocolitis; complete T-cell depletion in bone marrow and spleen	Necrotic enterocolitis
	Whole exome sequencing		*MYL2*	P^b^	PVS1, PM2, PP1, PM4	<1 year	Hypotonia, slow feeding	Not performed	Cardiomyopathy
			*SMAD9*	P	PVS1, PM2, PP5	1–6 years	Pre- and dysmaturity	Severe bronchiolitis with eosinophilia and edema	Rhinovirus bronchiolitis with pulmonary hypertension
			*MYBPC3*	P^b^	PVS1, PS3, PM2, PM3, PP1 and PVS1, PM2, PM3	<1 year	Dysmaturity	Hypertrophic cardiomyopathy	Hypertrophic cardiomyopathy
	Mitochondrial DNA analysis		*MT-ATP6*	P	PS4, PM6, PS3, PP1, PM2, PP3	1–6 years	Unspecified developmental disorder	Severe degeneration and loss of neurons in basal nuclei, thalamus and dentate nucleus; histologically suggestive for Leigh syndrome	Leigh syndrome
C	Panel	Arrhythmias	*KCNQ1*	P	PS3, PS4, PM2, PM5, PP3	<1 year	None	Not performed	Arrhythmia following infection
			*SCN5A*	P	PS3, PS4, PM5, PP1, PP3	6–12 years	None	No findings	Arrhythmia

^a^Methods and panels not listed did not yield any variants that were contributive for determining the cause of the sudden death

^b^Compound heterozygotic or homozygotic variant

^c^*LP* likely pathogenic, *P* pathogenic, *VUS* variant of unknown significance

In Group B, where standard PESUDIC findings were non-contributory but comorbidity was present, 73% of cases underwent one or more gene panels, 46% received a single nucleotide polymorphism (SNP) array, and 36% underwent open WES. Panels selected were primarily for arrhythmias, multiple congenital anomalies, and cardiomyopathies. A trio/duo panel or open WES analysis was performed in 6 cases (55%). Overall, one or more variants were identified in 7 cases (63%), with an LP/P variant found in 3 cases (27%). None of the identified variants were deemed contributory to determining the cause of death.

Group C, lacking both contributive findings and comorbidity, primarily underwent gene panels—especially those targeting arrhythmias and cardiomyopathies—with open WES performed in 6 cases (24%). In 11 cases (44%), a trio/duo panel or open WES analysis was included. Variants were identified in 14 cases (64%), and at least one LP/P variant was detected in 7 cases (28%). In 2 cases (8%), the identified variant was considered contributory to the cause of death, and both were related to cardiac pathways (Table 3).

There was no significant difference in the proportion of LP/P variants identified between infants (<1 year) and older children across all groups; LP/P variants were found in 26% of infants in Group A (*n* = 6/23), 40% in Group B (*n* = 2/11), and 20% in Group C (*n* = 4/20).

## Discussion

This study demonstrates the added value of postmortem genetic testing in the PESUDIC cohort. A (likely) pathogenic variant was identified in 23% of genetically tested cases, with the highest yield (28%) observed among those without contributive findings from standard postmortem examinations. This underscores the relevance of genetic testing, particularly when conventional diagnostics fail to identify a cause of death. In 13% of cases, the identified variants directly contributed to determining the cause of death, involving both cardiac (8%) and non-cardiac (5%) etiologies.

To better understand which cases benefit most from genetic testing, we categorized PESUDIC cases based on the presence of contributive findings and comorbidity. In Group A, where a cause of death had already been considered through standard diagnostics, an LP/P variant was found in 21% of cases and was contributive in determining the cause of death in 17% of the group. These findings are consistent with previous studies showing that genetic testing may add diagnostic value even when autopsy or other examinations reveal abnormalities. For instance, one study found LP/P variants in 11% of children under 3 years awaiting autopsy, and another identified LP/P variants involving arrhythmia genes in 16% of Dutch SIDS cases with and without limited autopsy findings [6, 17]. 

The cases without findings from conventional diagnostics were divided into a group with comorbidity (group B) and one without comorbidity (group C) in our study, because of different genetic pathways potentially underlying comorbidity. However, most studies on autopsy-negative SIDS or SUDC do not specifically include or exclude cases with comorbidity. Therefore, to compare to the literature, we must combine the results of groups B and C. We determined a yield of LP/P variants of 24% for infants <1 year and 36% for older children, aligning with previously reported yields. LP/P variants are reportedly determined in 3–33% of sudden death cases, especially among older children [5, 6, 18–22]. In group C, the genetic findings in our study were considered contributive to the cause of death in one infant and one older child, representing 5% and 20% of cases in that age category. This aligns with Heathfield’s review reporting a median diagnostic contribution of 4% in SIDS and with later studies showing contributive findings in up to 20% of autopsy-negative SUDI cases [5, 12]. 

Group C (no contributive findings and no comorbidity) consisted of significantly younger children, likely reflecting an overrepresentation of SIDS cases. This is consistent with previous PESUDIC data showing that 24% of infant deaths were classified as SIDS [1]. By definition, SIDS involves a lack of diagnostic findings and known comorbidity. Group C in our study best reflects the population studied by Oshima et al*.*, who excluded congenital anomalies and identified pathogenic variants in 15% of autopsy-negative sudden deaths under 1.5 years old (25). We found a similar yield (20%) in Group C.

Differences in diagnostic yields published may be due to variations in testing strategy, selected population, or criteria for interpreting variant pathogenicity. A major challenge in interpreting genetic testing studies is the lack of standardization. The genetic tests used in postmortem evaluations differ widely in terms of gene content, sequencing technology, and analysis pipelines, limiting cross-study comparisons. Additionally, evolving sequencing technologies over time introduce variability in detection rates, complicating comparisons across cohorts and study periods. Overall, the yield of genetic testing in our cases might have been affected by the high proportion of cases with a sudden death in the family, suggesting genetic factors at play.

Our findings reaffirm that cardiac genetic disorders are a significant contributor to SUDI and SUDC. In our cohort, 8% of cases had a cause of death related to inherited cardiomyopathies or arrhythmias—possibly an underestimation, as not all cases were tested for cardiac causes. In 5% of cases, the cause of death based on genetic testing was linked to non-cardiac pathways such as metabolic, mitochondrial, immunologic, or pulmonary conditions. This highlights the need to consider a broader range of genetic causes beyond cardiac conditions. Previous studies have reported metabolic or mitochondrial causes in 1–7% of SUDI cases, and neurological or syndromic genetic causes in 5% of SUDC cases [5, 6, 23]. 

Interestingly, unlike findings from Koh et al*.*, who reported a higher prevalence of cardiac causes in infants under one year compared to older children, our data did not show such clustering [6]. This may be due to the small proportion of older children in the Koh et al*.* study.

It is important to note that not all identified LP/P variants were considered contributive to the cause of death because they were secondary findings. Also, originally one VUS in group B was considered contributive based on prediction models, but advances in the field have since made this interpretation unlikely. This underscores the importance of ongoing refinement in variant classification and cautious interpretation to avoid over- or underestimating clinical significance. Prior research has shown that the diagnostic yield of genetic testing increases with the number of genes analysed [5]. While open WES provides broad coverage, it is resource-intensive and carries the risk of incidental or uncertain findings. Nevertheless, the benefits of identifying familial risk through cascade testing may outweigh the potential harms.

As of 2023, the United Kingdom’s National Health Service (NHS) has adopted a comprehensive postmortem gene panel comprising over 1200 genes relevant to SIDS and SUDC, including those related to epilepsy, arrhythmias, cardiomyopathies, and inborn errors of metabolism [24]. In contrast, the gene panel used by Koh et al*.* includes only 294 genes, with 68% overlap with the NHS panel [6]. In our study, 8 out of 14 contributive variants would have been missed using Koh’s panel, whereas only one (*SMAD9*) was not included in the NHS panel—highlighting the diagnostic advantage of broader gene coverage.

### Strengths and limitations

This study’s strengths include the use of a national, well-characterized cohort and the structured PESUDIC protocol, which enabled standardized data collection and clear subgroup analysis. To our knowledge, this is the first study to report the yield of postmortem genetic testing across specific clinical subgroups of SUDI and SUDC. However, limitations exist. Not all PESUDIC cases underwent the full diagnostic work-up, as postmortem investigations were subject to parental consent. This may have led to incomplete data and potential misclassification. Additionally, genetic testing was not performed systematically, and testing methods varied. Variant classification was based on the evidence available at the time of testing and may differ from current interpretations. These factors could have introduced selection bias and affected yield estimates. Although the proportion of PESUDIC cases undergoing genetic testing has remained relatively stable since 2017, changes in practice over time could still influence results. Finally, due to privacy regulations, we lacked access to data on genetic or cardiac screening outcomes among relatives.

## Conclusion

This study underscores the importance of incorporating comprehensive genetic testing into the postmortem evaluation of sudden unexpected deaths in infants and children, particularly when routine investigations do not reveal a cause. A detailed family history, the use of family screening tools—such as cardiologic evaluations—and multidisciplinary teamwork are essential for guiding test selection and interpreting results. In the absence of clinical clues, broad genetic testing can be considered, encompassing both cardiac and non-cardiac genes associated with sudden death.

Our findings support the implementation of genetic testing following negative conventional postmortem findings, regardless of patient age or presence of comorbidity. Adoption of standardized, expandable gene panels—such as the NHS panel—could promote consistency and improve diagnostic yield across populations. A comprehensive postmortem approach like PESUDIC, combined with genetic testing, has the potential to uncover inherited causes of SUDI and SUDC, inform family risk, and improve prevention strategies.

## Supplementary Information

Below is the link to the electronic supplementary material.

ESM 1 Supplementary Material 1 (DOCX 72.3 KB)

## Data Availability

No datasets were generated or analysed during the current study.

## Abbreviations

DNA: Deoxyribonucleic acid
LP variant: Likely pathogenic variant
P variant: Pathogenic variant
PESUDIC: Postmortem evaluation of sudden unexpected death in infants and children
SIDS: Sudden infant death syndrome
SUDC: Sudden unexpected death in children
SUDI: Sudden unexpected death in infants
VUS: Variant of unknown significance
WES: Whole exome sequencing
