# Supplementary material for: Genetic evaluation of sudden unexpected death in infants and children
Source: Eur J Pediatr. 2025 Oct 9;184(11):670. doi: 10.1007/s00431-025-06490-1 (PMC12511225; doi:10.1007/s00431-025-06490-1)
Supplement: Supplementary file 1 — Supplementary Material 1 (DOCX 72.3 KB) [file 431_2025_6490_MOESM1_ESM.docx]

**B SUPPLEMENTAL MATERIAL**

| **Group A: standard PESUDIC examinations yielded contributive findings** | | | | | |  |
| --- | --- | --- | --- | --- | --- | --- |
| **Case** | **Age category** | **Comorbidity** | **PESUDIC findings** | **Genetic analysis** | **(L)P variants** | **Cause of death** |
| 1 | <1 year | Hypotonia, slow feeding | History: no feeding, troubled breathing Physical examination: no findings Biochemical analysis: no findings Microbiological analysis: no findings Whole-body imaging: no findings Autopsy: no findings | SNP array, gene panel metabolic disorders, open WES | MYL2 | Cardiomyopathy |
| 2 | <1 year | Prematurity | History: no findings Physical examination: no findings Biochemical analysis: not done Microbiological analysis: not done Whole-body imaging: no findings Autopsy: necrotizing enterocolitis; complete T-cell depletion in bone marrow and spleen | Gene panel immunodeficiency | ADA | Necrotic enterocolitis |
| 3 | 6-12 years | Developmental disorder due to partial deletion chromosome 14, hypothyroidism | History: no findings Physical examination: no findings Biochemical analysis: no findings Microbiological analysis: influenza B in nasopharynx Whole-body imaging: diffuse lung consolidations Autopsy: not done | Open WES | None | Bacterial superinfect following influenza infection |
| 4 | 1-6 years | Obesity, bronchial hyperresponsiveness | History: no findings Physical examination: no findings Biochemical analysis: no findings Microbiological analysis: rotavirus in feces Whole-body imaging: no findings Autopsy: no findings | Open WES | None | Arrhythmia or SUDEP following rotavirus infection |
| 5 | 1-6 years | None | History: vomiting, diarrhea, decreased urination Physical examination: no findings Biochemical analysis: high urea in blood Microbiological analysis: adenovirus in feces Whole-body imaging: no findings Autopsy: no findings | Gene panels metabolic and mitochondrial disorders | None | Dehydration from gastroenteritis |
| 6 | <1 year | 22q11 deletion, pre- and dysmaturity | History: central apneas, saturation dip registered Physical examination: no findings Biochemical analysis: no findings Microbiological analysis: no findings Whole-body imaging: bilateral peribronchial infiltrative abnormalities in lungs Autopsy: not done | Gene panel multiple congenital anomalies, open WES | None | Obstructive apnea from upper respiratory infection |
| 7 | <1 year | None | History: abnormal temperature Physical examination: no findings Biochemical analysis: no findings Microbiological analysis: group B streptococcus in blood and cerebral spinal fluid Whole-body imaging: no findings Autopsy: bilateral pneumonia with neutrophilic granulocytes in lungs, liver and brain parenchyma | SNP array, gene panel multiple congenital anomalies | SERPINA1 POLG ALG1 | Group B streptococcus sepsis and meningitis |
| 8 | <1 year | None | History: no findings Physical examination: no findings Biochemical analysis: low glucose in blood, cerebral spinal fluid and vitreous humor; low CACT activity Microbiological analysis: no findings Whole-body imaging: no findings Autopsy: not done | SNP array, sequencing SLC25A20 | None^a^ | Hypoglycemia from CACT deficiency |
| 9 | ≥12 years | None | History: headache Physical examination: no findings Biochemical analysis: no findings Microbiological analysis: no findings Whole-body imaging: cerebral bleed Autopsy: cerebral bleed due to vascular malformation | Sequencing HHT genes | None | Brain swelling from cerebral bleed of vascular malformation |
| 10 | 1-6 years | None | History: chickenpox, abnormal temperature Physical examination: dried chickenpox lesions Biochemical analysis: no findings Microbiological analysis: varicella zoster virus on skin Whole-body imaging: no findings Autopsy: diffuse lymphocytic myocarditis | Sequencing KCNQ1, KCHNH2, LMNA, SCN5A, RYR2 and PLN | None | Arrhythmia following varicella zoster myocarditis |
| 11 | 1-6 years | None | History: blood vomiting, fever  Physical examination: no findings Biochemical analysis: no findings Microbiological analysis: no findings Whole-body imaging: consolidations right lung Autopsy: lung bleed and signs of severe chronic active infection in right lung | Sequencing COL3A1 | None | Lung bleed following pneumonia |
| 12 | 1-6 years | None | History: fever Physical examination: no findings Biochemical analysis: no findings Microbiological analysis: group A streptococcus in blood and throat Whole-body imaging: no findings Autopsy: not done | Gene panel sudden cardiac death | None | Group A streptococcus sepsis |
| 13 | 1-6 years | None | History: collapse during play Physical examination: no findings Biochemical analysis: no findings Microbiological analysis: no findings Whole-body imaging: pericardial fluid Autopsy: HHV-6 infection in pericardial fluid, signs of infection in lungs, myocardium and liver | Gene panels arrhythmias and cardiomyopathy | None | Arrhythmia following HHV-6 myocarditis |
| 14 | 6-12 years | None | History: long period of fatigue Physical examination: no findings Biochemical analysis: no findings Microbiological analysis: EBV in blood Whole-body imaging: more prominent heart ventricles, some pericardial fluid Autopsy: lymphocytosis in myocardium, EBV in myocardium, lungs and pleural fluid | Gene panels arrhythmias and immunodeficiency | None | Arrhythmia following EBV myocarditis |
| 15 | <1 year | Pre- and dysmaturity | History: no findings Physical examination: no findings Biochemical analysis: no findings Microbiological analysis: CMV in blood and pericardial fluid Whole-body imaging: increased pericardial fluid Autopsy: signs of myocarditis, CMV on myocardium | Gene panels arrhythmias, cardiomyopathy and immunodeficiency | None | Myocarditis following acute CMV infection |
| 16 | <1 year | None | History: no findings Physical examination: no findings Biochemical analysis: no findings Microbiological analysis: no findings Whole-body imaging: no findings Autopsy: congenital alveolar dysplasia and signs of myocardial infarct | Gene panel multiple congenital anomalies, open WES | None | Congenital alveolar dysplasia |
| 17 | ≥12 years | None | History: no findings Physical examination: no findings Biochemical analysis: no findings Microbiological analysis: no findings Whole-body imaging: no findings Autopsy: signs of lymphocytic myocarditis | Gene panel arrhythmias | SCN5A | Arrhythmia following viral myocarditis |
| 18 | <1 year | None | History: dyspnea/coughing Physical examination: no findings Biochemical analysis: no findings Microbiological analysis: no findings Whole-body imaging: bilateral lung consolidations and pleural fluid Autopsy: pleural fluid, signs of chronic aspiration and bacterial superinfection | Gene panel arrhythmias | None | Aspiration |
| 19 | ≥12 years | None | History: sudden fall on head Physical examination: no findings Biochemical analysis: not done Microbiological analysis: not done Whole-body imaging: extensive subdural bleed Autopsy: acute subdural bleed, subarachnoid bleed, contusions cerebrum, signs of herniation | Sequencing CACNA1A | None | Subdural bleed following fall on head |
| 20 | 1-6 years | None | History: fever Physical examination: no findings Biochemical analysis: no findings Microbiological analysis: enterovirus in blood, throat and feces and parainfluenza type 3 virus in throat Whole-body imaging: extensive bilateral lung consolidations Autopsy: signs of viral infection in lungs, liver, lymph nodes and spleen | Gene panels arrhythmias, cardiomyopathy and epilepsy | None | Convulsion following viral infection |
| 21 | <1 year | None | History: common cold, dyspnea/coughing Physical examination: no findings Biochemical analysis: no findings Microbiological analysis: no findings Whole-body imaging: bilateral lung consolidations, pleural fluid, inhomogeneous spleen Autopsy: dilated heart with T-cell and histiocyte infiltration in the myocardium and with focal cardiomyocyte necrosis | Gene panel cardiomyopathy | None | Viral myocarditis |
| 22 | <1 year | None | History: no findings Physical examination: no findings Biochemical analysis: low hemoglobin Microbiological analysis: no findings Whole-body imaging: large hyperacute intraperitoneal bleed originating from spleen Autopsy: abdominal bleed, ruptured spleen capsule with large blood clot | Gene panels coagulation disorders and metabolic disorders | None | Spleen bleed following birth |
| 23 | ≥12 years | None | History: no findings Physical examination: no findings Biochemical analysis: no findings Microbiological analysis: no findings Whole-body imaging: lung edema, enlarged right atrium and liver Autopsy: dilated heart, lymphocytic myocarditis | Gene panels arrhythmias and cardiomyopathy | None | Lymphocytic myocarditis |
| 24 | ≥12 years | Asthma | History: no findings Physical examination: no findings Biochemical analysis: no findings Microbiological analysis: no findings Whole-body imaging: no findings Autopsy: acute thrombus in left coronary artery | Sequencing LDLR, gene panel coagulation disorders | None | Myocardial infarct following atherosclerosis |
| 25 | <1 year | Prematurity | History: no findings Physical examination: no findings Biochemical analysis: no findings Microbiological analysis: E. Coli in blood and throat Whole-body imaging: diffuse lung consolidations and pleural fluid on the right Autopsy: not done | Gene panel immunodeficiency | None | Severe infection, possibly E. Coli |
| 26 | ≥12 years | Anorexia nervosa | History: decreased intake of food Physical examination: cachectic appearance Biochemical analysis: low blood sodium and high phosphate Microbiological analysis: no findings Whole-body imaging: no findings Autopsy: not done | Gene panel arrhythmias | None | Severe underweight due to anorexia nervosa |
| 27 | 6-12 years | Hypospadias, incontinence | History: fever Physical examination: no findings Biochemical analysis: no findings Microbiological analysis: parvovirus in blood Whole-body imaging: enlarged heart with dilated atria Autopsy: in both ventricles extensive abnormalities with cardiomyocyte necrosis, macrophages, lymphocytes and neutrophilic granulocytes; signs of viral infection in liver, lungs and thymus | Sequencing TTN and FLNC, gene panels arrhythmias and cardiomyopathy | None | Viral myocarditis |
| 28 | ≥12 years | None | History: pain Physical examination: no findings Biochemical analysis: not done Microbiological analysis: not done Whole-body imaging: cerebral venous thrombosis with hemorrhagic infarcts and herniation Autopsy: not done | Sequencing PROC, PROS1, SERPINC1, gene panel coagulation disorders | None | Cerebral venous thrombosis |
| 29 | 6-12 years | Albinism | History: fever, vomiting/diarrhea, pain Physical examination: no findings Biochemical analysis: CRP 100 mg/l Microbiological analysis: group A streptococcus in throat Whole-body imaging: induration of mesenterial fat tissue with multiple lymph nodes Autopsy: no findings on lung biopsy | Sequencing LYST, MYO5A, RAB27A, MLPH, BLOC1S6, HPS1, AP3B1, HPS6, HPS4, HPS5, DTNBP1, HPS3, AP3D1, BLOC1S3, BLOC1S5 and TERC, gene panel immunodeficiency | None | Gastrointestinal infection |
| 30 | 6-12 years | Unspecified developmental disorder, prematurity | History: fever, vomiting/diarrhea, dyspnea/coughing Physical examination: no findings Biochemical analysis: no findings Microbiological analysis: Haemophilus influenzae in blood Whole-body imaging: signs of bilateral pneumonia, pericarditis and encephalitis Autopsy: extensive bilateral pneumonia, Haemophilus influenzae in lungs | Gene panels mitochondrial disorders and multiple congenital anomalies | None | Pneumonia and sepsis from Haemophilus influenzae infection |
| 31 | ≥12 years | Unspecified developmental disorder, atrial flutter | History: sudden collapse during walk Physical examination: no findings Biochemical analysis: no findings Microbiological analysis: no findings Whole-body imaging: no findings Autopsy: hypertrophy and severe lipomatosis of right ventricular wall suggesting arrhythmogenic right ventricular dysplasia | Gene panels arrhythmias and cardiomyopathy | None | Arrhythmogenic right ventricular dysplasia |
| 32 | <1 year | Transposition of the great arteries | History: low temperature, dyspnea/coughing, fatigue Physical examination: no findings Biochemical analysis: no findings Microbiological analysis: no findings Whole-body imaging: no findings Autopsy: multiple myocardial infarcts in left and right ventricle | Gene panels multiple congenital anomalies, arrhythmias, cardiomyopathy and Noonan/RASopathy | None | Heart failure following myocardial infarcts |
| 33 | 1-6 years | Pre- and dysmaturity | History: fever, dyspnea/coughing Physical examination: no findings Biochemical analysis: no findings Microbiological analysis: no findings Whole-body imaging: signs of viral lung infection  Autopsy: severe active transmural bronchiolitis and extensive lung edema | Open WES | SMAD9 | Bronchiolitis possibly worsened by pulmonary hypertension |
| 34 | <1 year | None | History: drowsy, low intake Physical examination: no findings Biochemical analysis: unmeasurable low glucose in blood Microbiological analysis: no findings Whole-body imaging: no findings Autopsy: diffuse steatosis on liver biopsy | Gene panel multiple congenital anomalies | HMGCL | Hypoglycemia from HMG-CoA lyase deficiency |
| 35 | ≥12 years | None | History: long period of fatigue, leg pain, recent spontaneous hematomas, dyspnea Physical examination: no findings Biochemical analysis: high urea and creatinine in blood Microbiological analysis: no findings Whole-body imaging: bilateral small kidneys Autopsy: atrophic kidneys with signs of chronic pyelonephritis, focal segmental sclerosis and glomerulonephritis | SNP array, gene panel abnormality of the genitourinary system | None | Terminal kidney failure |
| 36 | <1 year | Hirschprung | History: vomiting/diarrhea and low intake Physical examination: no findings Biochemical analysis: no findings Microbiological analysis: group A streptococcus in blood and throat; enterovirus in throat and feces Whole-body imaging: no findings Autopsy: no findings | SNP array, sequencing PHO2B, SOX10, EDNRB and PHOX2B, open WES | None | Enterovirus meningitis with group A streptococcus superinfection |
| 37 | ≥12 year | None | History: frequent urination, increased thirst, weight loss  Physical examination: ketone smell Biochemical analysis: high glucose in blood, spinal fluid and vitreous humor; positive islet antigen-2 antibodies Microbiological analysis: no findings Whole-body imaging: no findings Autopsy: pancreatic islets without insulin and with lymphohistiocytic infiltrate; Armanni-Ebstein lesions in kidneys | Open WES | None | Diabetic ketoacidosis |
| 38 | 1-6 years | None | History: dyspnea/coughing and fatigue Physical examination: no findings Biochemical analysis: high NT-proBNP in blood Microbiological analysis: no findings Whole-body imaging: prominent left ventricle wall, pericardial fluid  Autopsy: not done | SNP array, gene panel cardiomyopathy | None | Cardiomyopathy |
| 39 | 1-6 years | Prematurity, esophageal atresia | History: fever, vomiting and fatigue Physical examination: vomit on mouth Biochemical analysis: no findings Microbiological analysis: no findings Whole-body imaging: trachea filled with vomit Autopsy: vomit in trachea and bronchi with subacute/chronic inflammation in trachea | SNP array, open WES | None | Aspiration |
| 40 | ≥12 years | None | History: heart palpitations on day of death Physical examination: no findings Biochemical analysis: no findings Microbiological analysis: no findings Whole-body imaging: no findings Autopsy: no findings | SNP array, gene panels arrhythmias and cardiomyopathy, open WES | None | Arrhythmia |
| 41 | <1 year | None | History: agitation Physical examination: no findings Biochemical analysis: no findings Microbiological analysis: no findings Whole-body imaging: no findings Autopsy: volvulus of small intestine based on non-rotation of the intestines | SNP array | None | Volvulus following congenital malformation |
| 42 | ≥12 years | None | History: sudden death during exercise Physical examination: no findings Biochemical analysis: no findings Microbiological analysis: no findings Whole-body imaging: no findings Autopsy: not done | Sequencing TTN, gene panels arrhythmias and cardiomyopathy | None | Arrhythmia |
| 43 | 6-12 years | Heart murmur | History: fever, vomiting/diarrhea, dyspnea/coughing, low intake, sweating, leg pain Physical examination: no findings Biochemical analysis: high CRP in blood Microbiological analysis: influenza B in throat Whole-body imaging: increased pericardial fluid, diffuse thickened myocardium Autopsy: not done | Sequencing TTN, gene panel cardiomyopathy | None | Myocarditis |
| 44 | 1-6 years | None | History: no findings Physical examination: no findings Biochemical analysis: no findings Microbiological analysis: no findings Whole-body imaging: enlarged and thickened heart Autopsy: extremely enlarged heart with hypertrophy and degenerative abnormalities in the cardiomyocytes | SNP array, gene panel multiple congenital anomalies, mitochondrial DNA analysis | MRPL44 | Hypertrophic cardiomyopathy |
| 45 | 1-6 years | Hirschprung | History: vomiting/diarrhea and low intake Physical examination: sunken eyes Biochemical analysis: no findings Microbiological analysis: enterovirus and norovirus in feces Whole-body imaging: no findings Autopsy: involution of thymus indicative of severe illness since a couple days; ischemia in intestines | SNP array, sequencing PHOX2B, SOX10 and EDNRB | None | Dehydration following viral infection |
| 46 | 1-6 years | None | History: fever, vomiting/diarrhea, fatigue Physical examination: no findings Biochemical analysis: no findings Microbiological analysis: enterovirus in throat and feces Whole-body imaging: no findings Autopsy: chronic inflammation and destruction of cardiomyocytes, most likely viral origin | Sequencing SERPINA1 and TTN, gene panel cardiomyopathy | None | Enterovirus myocarditis |
| 47 | 1-6 years | SMA type 1 | History: sudden death during transport Physical examination: no findings Biochemical analysis: no findings Microbiological analysis: no findings Whole-body imaging: no findings Autopsy: no findings | SNP array, sequencing TTN | None | Arrhythmia related to SMA |
| 48 | ≥12 years | Dysmaturity | History: no findings Physical examination: no findings Biochemical analysis: no findings Microbiological analysis: no findings Whole-body imaging: no findings Autopsy: enlarged heart, thickened left ventricle and septum, abnormalities in intercalated discs | SNP array, gene panels arrhythmias and cardiomyopathy, open WES | None | Arrhythmia |
| 49 | 1-6 years | Unspecified developmental disorder | History: worse motor skills, muscle weakness Physical examination: bilateral ptosis Biochemical analysis: no findings Microbiological analysis: no findings Whole-body imaging: abnormalities in basal ganglia and white matter in mesencephalon, pons and medulla oblongata Autopsy: severe degeneration and loss of neurons in basal nuclei, thalamus and dentate nucleus; histologically suggestive for Leigh syndrome | SNP array, mitochondrial DNA analysis, open WES | Mt-ATP6 | Leigh syndrome |
| 50 | 1-6 years | None | History: fever, blue lips Physical examination: no findings Biochemical analysis: high CRP in blood Microbiological analysis: Kingella kingae in spinal fluid Whole-body imaging: large amount of pericardial fluid Autopsy: endocarditis of aortic valve with rupture of the aortic wall | Gene panel immunodeficiency | None | Kingella kingae endocarditis resulting in aorta wall rupture |
| 51 | <1 year | None | History: abnormal breathing pattern Physical examination: no findings Biochemical analysis: no findings Microbiological analysis: no findings Whole-body imaging: no findings Autopsy: abnormal lung maturation | SNP array, sequencing BCLAF1, BTBD7, FGF10, FGFR2, FOXF1, TBX4 and TCF21, mitochondrial DNA analysis | None | Congenital alveolar dysplasia |
| 52 | <1 year | None | History: chickenpox, fever, atypical febrile seizure Physical examination: chickenpox rash Biochemical analysis: high CRP in blood Microbiological analysis: group A streptococcus in spinal fluid Whole-body imaging: bilateral consolidations in lungs Autopsy: extensive necrotizing vasculitis in lungs and liver; group A streptococcus in lung and spleen biopsy | Gene panels nephrotic syndrome and immunodeficiency | None | Group A streptococcus sepsis |
| 53 | 6-12 years | None | History: fever and leg pain Physical examination: no findings Biochemical analysis: high CRP in blood Microbiological analysis: group A streptococcus in blood and throat Whole-body imaging: not done Autopsy: thymus, liver and spleen show abnormalities indicative of sepsis | Mitochondrial DNA analysis, open WES | None | Group A streptococcus sepsis |
| 54 | <1 year | None | History: no findings  Physical examination: no findings Biochemical analysis: no findings Microbiological analysis: no findings Whole-body imaging: enlarged liver Autopsy: slightly dilated right ventricular wall and very low proportion of dystrophin in heart tissue | Gene panels arrhythmias and cardiomyopathy, open WES | DMD | Cardiomyopathy |
| 55 | 1-6 years | None | History: no findings Physical examination: no findings  Biochemical analysis: no findings Microbiological analysis: group A streptococcus in blood, spinal fluid and throat Whole-body imaging: no findings Autopsy: severe chronic bronchitis | Sequencing PTEN, gene panel immunodeficiency | PTEN | Fulminant group A streptococcus infection following chronic bronchitis |
| 56 | ≥12 years | None | History: no findings Physical examination: no findings Biochemical analysis: no findings Microbiological analysis: no findings Whole-body imaging: no findings Autopsy: enlarged heart with fibrosis and a prominent right ventricular wall | Gene panel cardiomyopathy, open WES | PRKAG2 | Arrhythmia |
| 57 | <1 year | Dysmaturity | History: low intake, agitation Physical examination: no findings Biochemical analysis: no findings Microbiological analysis: no findings Whole-body imaging: enlarged heart with hypertrophic left ventricle Autopsy: hypertrophic cardiomyopathy | SNP array, sequencing KCNJ11 and ABCC8, open WES | MYBPC3 MYBPC3 | Hypertrophic cardiomyopathy |
| 58 | <1 year | Pre- and dysmaturity | History: agitation Physical examination: no findings Biochemical analysis: no findings Microbiological analysis: not done Whole-body imaging: subdural hematoma Autopsy: subdural and intraparenchymal bleeds | Open WES, determination of zygosity | None | Intracranial bleeds |
| 59 | 1-6 years | Prematurity | History: subglottic laryngitis diagnosed Physical examination: no findings Biochemical analysis: no findings Microbiological analysis: group A streptococcus in blood and throat Whole-body imaging: bilateral pulmonary consolidations Autopsy: viral bronchiolitis, signs of stress or viral myocarditis; group A streptococcus in lung biopsy | Determination of zygosity | None | Group A streptococcus superinfection following subglottic laryngitis |
| 60 | 1-6 years | None | History: fever, vomiting/diarrhea, no urination Physical examination: not reported Biochemical analysis: not done Microbiological analysis: adenovirus in feces Whole-body imaging: no findings Autopsy: fulminant viral myocarditis and signs of enterocolitis | SNP array, gene panel immunodeficiency | None | Adenovirus myocarditis and gastroenteritis |
| 61 | ≥12 years | Asthma | History: in weeks before death near-collapse Physical examination: no findings Biochemical analysis: no findings Microbiological analysis: no findings Whole-body imaging: no findings Autopsy: signs of hypertrophic cardiomyopathy and viral lymphocytic myocarditis | Gene panel cardiomyopathy | None | Arrhythmia following lymphocytic myocarditis and possible underlying cardiomyopathy |
| 62 | <1 year | None | History: rhinitis, otitis, vomiting/diarrhea, dyspnea/coughing Physical examination: no findings Biochemical analysis: no findings Microbiological analysis: Haemophilus influenzae in blood; group A streptococcus in throat, urine and ear secretion Whole-body imaging: no findings Autopsy: not done | Gene panels metabolic disorders and sudden cardiac death | None | Bacterial superinfection following viral infection |
| 63 | 1-6 years | None | History: dyspnea/coughing, low intake and sweating Physical examination: no findings Biochemical analysis: no findings Microbiological analysis: no findings Whole-body imaging: increased pericardial and pleural fluid and diffuse lung edema | Gene panel sudden cardiac death | None | Unspecified heart failure |
| 64 | ≥12 years | Prematurity | History: extreme fatigue Physical examination: not reported Biochemical analysis: no findings Microbiological analysis: no findings Whole-body imaging: no findings Autopsy: signs of recent cardiac ischemia, diffuse leukocytosis in myocardium and pericardium | Gene panel sudden cardiac death | KCNE1 | Viral pericarditis |
| 65 | <1 year | None | History: fall on head followed by status epilepticus  Physical examination: not reported Biochemical analysis: no findings Microbiological analysis: no findings Whole-body imaging: bilateral symmetric cytotoxic edema in cerebrum Autopsy: signs of cerebral herniation | Sequencing POLG | None | Hypoxic encephalopathy following status epilepticus |
| 66 | <1 year | None | History: no findings Physical examination: no findings Biochemical analysis: not done Microbiological analysis: no findings Whole-body imaging: no findings Autopsy: extensive necrosis of myocardium with infiltration from lymphocytes | Open WES | None | Lymphocytic myocarditis |
|  | | | | | | |
| **Group B: standard PESUDIC examinations yielded no contributive findings but comorbidity was present** | | | | | | |
| **Case** | **Age category** | **Comorbidity** | **Standard PESUDIC examinations performed** | **Genetic analysis** | **(L)P variants** | **Cause of death** |
| 67 | 1-6 years | Hydronephrosis, febrile seizures | History, physical examination, biochemical analysis, microbiological analysis, whole-body imaging | SNP array | None | Unexplained |
| 68 | <1 year | Dysmaturity | History, physical examination, biochemical analysis, microbiological analysis, whole-body imaging, autopsy | SNP array, gene panel multiple congenital anomalies, open WES | BCS1L | Unexplained |
| 69 | 1-6 years | Frequent upper airway infections, febrile seizure, poor growth | History, physical examination, biochemical analysis, microbiological analysis, whole-body imaging | SNP array, gene panel metabolic disorders, open WES | None | Unexplained |
| 70 | 1-6 years | Prematurity | History, physical examination, biochemical analysis, microbiological analysis, whole body imaging | SNP array, gene panels multiple congenital anomalies and mitochondrial disorders, open WES | COL6A2 | Unexplained |
| 71 | <1 year | Prematurity | History, physical examination, biochemical analysis, microbiological analysis, whole body imaging | SNP array, gene panel multiple congenital anomalies | CFHR5 | Unexplained |
| 72 | <1 year | Prematurity | History, physical examination, biochemical analysis, microbiological analysis, whole body imaging | Gene panel arrhythmias | None | Unexplained |
| 73 | ≥12 years | Unspecified developmental disorder | History, physical examination, biochemical analysis, microbiological analysis, whole body imaging, autopsy | Gene panels arrhythmias, cardiomyopathy, multiple congenital anomalies and thoracic aortic aneurysm and dissection | None | Arrhythmia |
| 74 | ≥12 years | Developmental disorder with epilepsy | History, biochemical analysis, microbiological analysis, autopsy | Sequencing PKP2, DSG2, DSC2, JUP, DSP, TGFbeta3 and RYR2, gene panels arrhythmias and cardiomyopathy | None | Unexplained |
| 75 | <1 year | Prematurity, cleft lip and palate | History, physical examination, biochemical analysis, microbiological analysis, whole body imaging, autopsy | Sequencing PKP2, DSG2, DSC2, JUP, DSP, RYR2 and TGFbeta3, gene panels arrhythmias and cardiomyopathy | None | Unexplained |
| 76 | <1 year | Neonatal thrombocytopenia | History, physical examination, biochemical analysis, microbiological analysis, whole body imaging | Sequencing NPC1, NPC2 | None | Unexplained |
| 77 | 1-6 years | Smith-Magenis syndrome | History, physical examination, biochemical analysis, microbiological analysis, whole body imaging, autopsy | Open WES | None | Unexplained |
| ^a^ Heterozygotic variant in SLC25A20 determined homozygotic in cDNA suggesting pathogenic second variant | | | | | | |
